# Decline in prevalence of congenital sensorineural deafness in Dalmatian dogs in the United Kingdom

**DOI:** 10.1111/jvim.15776

**Published:** 2020-06-16

**Authors:** Tom Lewis, Julia Freeman, Luisa De Risio

**Affiliations:** ^1^ The Kennel Club London UK; ^2^ School of Veterinary Medicine and Science The University of Nottingham Leicestershire UK; ^3^ Animal Health Trust Newmarket UK

**Keywords:** brainstem auditory evoked response, Dalmatians, hearing function, inherited

## Abstract

**Background:**

Congenital sensorineural deafness (CSD) is the most common type of deafness in Dalmatian dogs.

**Objectives:**

To use results of CSD screening in Dalmatian dogs in the United Kingdom in genetic analysis and to determine any changes in the prevalence of CSD in this breed over time.

**Animals:**

A total of 8955 Dalmatian puppies undergoing hearing function screening using brainstem auditory evoked response (BAER) between July 1992 and February 2019.

**Methods:**

Results of BAER testing and pigmentation phenotypic data were linked to the UK Kennel Club Dalmatian pedigree database. Mixed model analysis was used to estimate variance parameters.

**Results:**

The overall prevalence of CSD was 17.8% (13.4%, unilateral; 4.4%, bilateral). Heritability of CSD was approximately 0.3 (across models) and significantly >0. Genetic correlations between CSD and blue irises (+0.6) and pigmented head patch (−0.86) were large in magnitude and significantly different form 0. Significant improving phenotypic and genetic trends were identified, likely as the result of selection against deafness, equivalent to avoiding breeding with the 4% to 5% of animals with the highest genetic risk of CSD.

**Conclusions and Clinical Importance:**

A decrease in the prevalence and genetic risk of CSD implies breeders have been selecting for hearing dogs. Selective breeding based on estimated breeding values (EBVs) can help further decrease the prevalence of CSD in Dalmatians in the future.

**Abbreviations**
AHT: animal health trust
BAER: brainstem auditory evoked response
CSD: congenital sensorineural deafness
EBVs: estimated breeding values
*h*^2^: heritability
KC: Kennel Club
*r*_A_: additive genetic correlation
UK: United Kingdom

## 1 INTRODUCTION

Congenital sensorineural deafness (CSD) is the most frequent type of deafness in dogs and results from loss of hearing receptors in the first 3 to 4 weeks after birth, and the condition is permanent. It can be objectively and noninvasively diagnosed by brainstem auditory evoked response (BAER) testing.^1^, ^2^

The Dalmatian is the breed with the highest reported prevalence of CSD being as high as 30% (8%, bilateral; 22%, unilateral) in the United States.^3^ The only study conducted in the United Kingdom (UK) reported a CSD prevalence in Dalmatians of 18.4% (13.1%, unilateral; 5.3%, bilateral).^4^ Dalmatian puppies with a pigmented coat patch at birth have a lower prevalence of CSD than those without a patch, and puppies with blue irises have a higher prevalence of CSD than puppies with brown irises.^3^, ^5^, ^6^, ^7^ Genetic correlations between these pigmentation phenotypes and CSD have been identified.^5^, ^6^, ^7^

The heritability estimates of CSD in Dalmatians ranged from 0.27 to 0.76 in studies conducted in the United States, Germany, and Switzerland.^5^, ^6^, ^7^, ^8^, ^9^ The inheritance of CSD appears to be complex, and its genetic basis remains unknown despite efforts to identify causative variants.^10^, ^11^ Dogs with normal hearing status (based on BAER testing) can be carriers of the risk mutations responsible for CSD, and therefore disease prevention by selective breeding is challenging.

The magnitude of the range of heritability estimates indicates that estimated breeding values (EBVs) could improve the accuracy of selection. Where the narrow sense heritability is equal to 1, all phenotypic variation is additive genetic variance, and individuals' phenotypes reflect exactly their genetic liability. Conversely, when the heritability is 0, there is no additive genetic variance underlying phenotypic variance, and extant phenotypic variation is comprised of other influential factors, such as environment. Between these boundary values, some additive genetic variance impacting the trait exists, but is overlaid by variation from other, non‐genetic influences (the heritability indicating to what extent), and phenotypes thus are a mix of individual genetic liability (which is inherited by offspring) and individual nonadditive genetic and environmental influences (which are not). Therefore, the phenotype is an imperfect guide to underlying genetics, and selection using phenotypes will be prone to an increasing degree of inaccuracy at lower heritability. The calculation of EBVs uses pedigree data to determine the degree of relationship among all animals evaluated and, in conjunction with phenotypic data (ie, BAER test results) from at least some individuals in the pedigree, to estimate the genetic liability for all individuals in the pedigree based on their own phenotype and those of all relatives. With sufficient data, EBVs will be a more accurate reflection of true underlying genetic liability or risk than phenotypes, and therefore a more accurate metric to use in selection.

Our aim was to estimate the heritability of CSD and, from the resultant breeding values and results of BAER screening, determine whether any changes in the genetic liability and prevalence of CSD have occurred in UK Kennel Club (KC) registered Dalmatians over the past 20 to 30 years.

## 2 MATERIALS AND METHODS

The study was approved by the institution's clinical research ethics committee. The institution's hearing clinic database was searched for purebred Dalmatian puppies that underwent BAER testing at 4 to 10 weeks of age between 29 June 1992 and 4 February 2019. The BAER testing was performed as part of a hearing‐screening program. Informed consent of the puppies' owners was obtained before BAER testing. Inclusion criteria were that BAER was carried out on all surviving puppies in the litter, phenotypic data were recorded, and UK KC registration details were available to link the phenotypic data to the KC pedigree database for quantitative genetic investigations.

### 2.1 Phenotypic data

Age, sex, presence of a pigmented coat patch or patches on the head or elsewhere on the body at birth, spot color, iris color, and hearing status based on BAER testing were recorded for each puppy. Spot color was categorized as black, liver, lemon, orange, or tricolor. Iris color was recorded for each eye as completely brown, completely blue, or partly blue. The hearing status of the puppies was classified (per ear) as “normal,” “deaf,” or “impaired/equivocal” (not fitting the profile of either “normal” or “deaf”), giving 9 bilateral classifications.

### 2.2 BAER testing protocol

As previously reported by the authors,^12^, ^13^, ^14^ BAER recordings were carried out using an electrodiagnostic machine (Sapphire 2ME 2‐channel system, Medelec, Oxford Instruments Medical, Old Woking, UK), Synergy N‐EP 5‐channel system (Medelec Viasys Healthcare, Warwick, UK) or Synergy EDX 2‐channel system (Optima Medical, Guildford, UK). The amplifier was set to 20 μV per division for the Sapphire 2ME and, for the Synergy N‐EP and the Synergy EDX, to 10 μV per division, and sweep duration was 10 ms for all 3 machines. Low‐frequency filter was 100 Hz, with a high frequency of 2 kHz for the Sapphire 2ME and 3 kHz for the Synergy N‐EP and Synergy EDX.

Stainless steel needle electrodes (12 mm long, 0.3 mm diameter) were placed subdermally at 3 sites on the head. The ground electrode was located over the occipital protruberance, the reference electrode over the vertex, and the recording electrode just in front of the tragus of the tested ear. Rarefaction or alternating clicks, 0.1 ms in duration, were presented at 80 dBnHL through an unshielded audiometric headphone (model TDH49P, Medelec with the Sapphire 2ME and Synergy N‐EP, model TDH39P, Nicolet with the Synergy EDX) held against the external ear opening. Before testing, puppies were allowed to become sleepy naturally, which, because of the minimally invasive nature of the test, enabled testing to be carried out without sedation or anesthesia. Data were acquired at a click rate of 20to 30/s and at least 512 responses were signal averaged to eliminate artifact. If a normal trace was absent at 80 dB, the test was repeated at 100 dB for the Sapphire 2ME and Synergy N‐EP, and 95 dB for the Synergy EDX. To exclude the contribution from a normal‐hearing contralateral ear, white noise, at 20 dB lower than the stimulus level, was delivered into the nonstimulated ear. Both ears were tested individually; first the right ear, followed by the left ear.

### 2.3 Pedigree

Phenotypic data records were linked to the UK KC Dalmatian pedigree database, using the unique KC registration number allocated to each registered individual or the parental KC numbers. Because not all puppies were registered with the KC before to the hearing test, some puppies and litters were identified by means of date of birth and their parents' KC registration numbers. The KC database includes registration number, registered name, sex, date of birth, sire registration name and number, and dam registration name and number. The pedigree data used in the analysis were the entire KC‐registered Dalmatian pedigree appended with puppies that had identifiable littermates or parents in the KC pedigree database.

### 2.4 Estimation of genetic parameters

Mixed linear models using ASREML^15^ were fitted to the hearing status data (0 = bilaterally normal, 1 = unilaterally deaf, 2 = bilaterally deaf, missing values excluded) of puppies to estimate variance components. The general form of the linear model was as follows:

Y=Xb+Za+Wc+e

where Y is the vector of observations, X, Z, and W are known incidence matrices, b is the vector of fixed effects, a is the vector of random additive genetic effects with the distribution assumed to be multivariate normal, with parameters (0, *σ* ^2^_a_ A), c is the vector of litter effects, and e is the vector of residuals distributed with parameters (0, *σ* ^2^_litt_I) and (0, *σ* ^2^_e_I), respectively. I is an identity matrix of the appropriate size, A is the additive genetic relationship matrix and *σ* ^2^ denotes the variance of each of the respective random effects. Preliminary models indicated that the inclusion of litter as a random effect in the mixed model analysis of hearing status was highly significant (*P* < .001, likelihood ratio test), but that additional inclusion of dam as a random effect was not (*P* > .05). The fixed effects included in the univariate model were sex, year of test, number of blue eyes, and presence of pigmented head patch. Age (in weeks) at test and inbreeding coefficient were included as covariates.

### 2.5 Liability transformation

An underlying, normally distributed liability of hearing status was assumed, and hearing status data (0/1/2) were transformed into the mean deviation of individuals with values exceeding truncation points based on the proportion of hearing, uni‐ and bilaterally deaf categories of data.^16^ Analyses were repeated using these liability values as the dependent variable. Where additional traits were included in bivariate analyses (see below), analyses using liability transformations of these traits also were performed.

### 2.6 Bivariate analysis

Bivariate analysis was performed with the dependent variables hearing status (separately on the observed and liability scale) and (a) the number of blue eyes (observed and liability scale) and (b) the presence of pigmented head patch (observed binary and liability scales) included in an animal + litter model, with fixed effects as previously described. The variances (*σ* ^2^) were replaced with the 2 × 2 variance/covariance matrix for both traits and the direct product operator.

### 2.7 Heritability and genetic correlation

The phenotypic variance, denoted as $\sigma_{p}^{2}$, comprises the sum of the additive genetic variance, litter variance, and residual variance ($\sigma_{a}^{2}+\sigma_{\text{litt}}^{2}+\sigma_{e}^{2}$). The heritability (*h* ^2^) is calculated as the proportion of the phenotypic variance explained by the additive genetic variance ($\sigma_{a}^{2}/\sigma_{p}^{2}$), and litter effect calculated as the proportion of phenotypic variance explained by litter variance ($\sigma_{\text{litt}}^{2}/\sigma_{p}^{2}$). In bivariate analyses, the additive genetic variance of each trait and the covariances between each pair of traits are used to calculate the genetic correlation:

$$r_{A\left(a,b\right)}=\frac{\sigma_{A\left(a,b\right)}}{\sqrt{\sigma_{Aa}^{2}\cdot \sigma_{Ab}^{2}}}$$

where *r*_A_ is the additive genetic correlation, *a* and *b* denote the 2 traits in question, $\sigma_{A}^{2}$ denotes the additive genetic variance of traits and *σ*_A(*a*,*b*)_ is the additive genetic covariance of trait *a* with *b*. Correlations of litter and residual effects were calculated similarly.

### 2.8 Assessment of phenotypic and genetic trends in CSD

The data were interrogated for any changes in prevalence of CSD, and in genetic liability of CSD, over year of birth, using linear regression. Prevalence of CSD (unilateral + bilateral) was the y‐variable regressed on the years of birth for which complete data were available (1993‐2018) to detect any trend in CSD over time. The presence of any genetic trend was determined by regression of mean EBV of dogs born per year (from a univariate analysis of hearing status on the observed scale) on year of birth (for years for which complete data were available; 1993‐2018). Linear regressions were performed using MATLAB.^17^ The genetic trend may be assumed to be a response to selection (*R*), and rearrangement of the equation: *R = i h* ^2^ *σ*_P_/*L* (where *L* is the approximate generation interval of 4 years) can be used to determine *i* (the mean deviation of individuals with phenotypic values exceeding the truncation point^16^), and thus an approximate selection intensity.

## 3 RESULTS

A total of 8955 puppies met the inclusion criteria. These comprised 1225 unique litters from 780 unique dams and 375 unique sires. There were 4499 (50.2%) males and 4456 (49.8%) females. The distribution of age at time of testing is given in Table 1. The data were evenly distributed over year of test with no observable changing trend, from n = 216 (2.41%) in 1993 to n = 519 (5.80%) in 2003, aside from the years 1992 and 2019 (n = 69 and 48, respectively) in which data were collected for only part of the year. Inbreeding coefficients ranged from 0 to 0.38 (median, 0.057; interquartile range, 0.074; 0.0307 to 0.1044).

**TABLE 1:** Age in weeks at test

Age at BAER test	n	Percentage
4 weeks (ie, ≥28 days)	629	7.02%
5 weeks	3984	44.49%
6 weeks	3497	39.05%
7 weeks	684	7.64%
8 weeks	115	1.28%
9 weeks (ie, ≥63 days)	46	0.51%
	8955	100.00%

*Note:* Distribution of age at time of brainstem auditory evoked response (BAER) test.

Of this overall data set, 7349 (82.1%) puppies had normal hearing status, 1201 (13.4%) had unilateral CSD (650 puppies in the right ear; 551 puppies in the left ear), 397 (4.4%) had bilateral CSD, and 8 (0.1%) had unilaterally impaired/equivocal hearing status (5 in the right ear and 3 in the left ear), which were excluded from analysis (Table 2). Puppies were classified as having impaired/equivocal hearing if there was a repeatable trace in either ear where the waveform was present, repeatable, and the amplitude and latency decreased or increased with decrease or increase of the stimulus, but the waveform amplitude and latency were not of the levels expected. In these cases, follow‐up BAER always was offered free of charge a few weeks later, but because the puppies by that stage were at their new homes, this offer was not taken up by the new owner. These 8 puppies were excluded from the ASREML analysis.

**TABLE 2:** Frequency of hearing status/deafness

Left\right	Equivocal	Hearing	Deaf	Total
Equivocal	0	3	0	3
Hearing	4	7349	650	8003
Deaf	1	551	397	949
	5	7903	1047	8955

*Notes:* Frequency of hearing status across left and right ear in n = 8955 puppies that met the inclusion criteria. Equivocal results (or “impaired” hearing) were excluded from analysis.

Iris color was bilaterally brown in 8598 (96%) puppies, 1 blue (completely) and 1 brown in 262 (3%) puppies, bilaterally (completely) blue in 65 (0.7%) puppies, and at least 1 partially blue in 30 (0.3%) puppies (Table 3). A pigmented coat patch was present on the head at the time of BAER test in 945 (10.6%) puppies and was absent in 8010 (89.4%) puppies. Of the 945 puppies with a pigmented coat patch, 941 (99.58%) had a patch on the head only and 4 (0.42%) had a patch on the head and a patch on the body. Spot color was black in 6807 (76%) puppies, liver in 2135 (23.9%), and other (lemon/orange/tricolor) in 13 (0.1%) puppies. The number, proportion, and liability transformation for each category within each dependent variable used in analysis (hearing status, number of brown/blue eyes and presence/absence of pigmented head patch) are given in Table 4.

**TABLE 3:** Frequency of iris color

Left\Right	Missing	Brown	Blue	Total
Missing	5	8	0	13
Brown	16	8598	157	8771
Blue	1	105	65	171
	22	8711	222	8955

*Notes:* Frequency of brown/blue iris color in left and right eyes in n = 8955 puppies that met the inclusion criteria. Puppies with partially blue irises were excluded from analysis and are termed here as “missing”.

**TABLE 4:** Variate category numbers and transformations

	Number	Proportion	Liability transformation
Bilaterally hearing	7349	0.821	−0.317
Unilaterally deaf	1201	0.134	1.240
Bilaterally deaf	397	0.044	2.116
Total	8947	1	
2 brown eyes	8598	0.963	−0.085
1 brown/blue eye	262	0.029	2.035
2 blue eyes	65	0.007	2.761
Total	8925	1	
No pigmented head patch	8010	0.894	−0.203
Pigmented head patch present	945	0.106	1.732
Total	8955	1	

*Note:* Number, proportion, and liability transformation for each category within each dependable variable used in analysis.

### 3.1 Univariate analysis of hearing status

Estimates of heritability of hearing status (CSD in 0/1/2 ears) on the observed and liability scale were 0.305 (SE [standard error of the effect] 0.0404) and 0.271 (SE 0.0379), respectively, and were significantly >0 (*P* < .001, likelihood ratio test). Smaller, but still significantly >0 (*P* < .001), litter variance effects on hearing status were estimated as 0.090 (SE 0.0123) and 0.089 (SE 0.0120) on the observed and liability scale, respectively. There was a small but significant effect (*P* < .01) of sex on hearing status on both scales, for example, of +0.041 (SE 0.0099) for females compared to males on the observed scale. There was a significant effect of age at test (in weeks) on hearing status (*P* < .01), largely because of the higher detected effect for dogs aged 8 and 9 weeks (+0.0197 [SE 0.0685] and +0.251 [SE 0.0979], respectively, on the observed scale compared to 7 weeks. None of the effects for other age categories showed significant differences. Presence of a patch on the head and number of blue eyes were significant factors (*P* < .01), with the patch negatively associated with deafness (−0.097 [SE 0.0165]) and 1 and 2 blue eyes positively associated with deafness; +0.330 [SE 0.0303] for 1 blue eye, and +0.481 [SE 0.0592] for 2 blue eyes (all effects reported on the observed scale compared to absence of patch or no blue eyes). No significant association was found between hearing status and the remaining fixed effects or covariates.

### 3.2 Bivariate analysis of hearing status and blue eyes/presence of head patch

Bivariate analysis of hearing status (CSD in 0/1/2 ears) with number of blue eyes on the observed (liability) scale yielded estimates of heritability of 0.330 (SE 0.0411) [0.292; SE 0.0384] for hearing status and 0.172 (SE 0.0276) [0.165; SE 0.0267] for number of blue eyes; litter effect of 0.095 (SE 0.0124) [0.094; SE 0.0122] for hearing status and 0.038 (SE 0.0091) [0.042; SE 0.0091] for number of blue eyes. Bivariate analysis of hearing status with the presence of pigmented head patch on the observed (liability) scale yielded estimates of heritability of 0.343 (SE 0.0406) [0.308; SE 0.0385] for hearing status and 0.082 (SE 0.0181) [0.078; SE 0.0179) for head patch; litter effect of 0.086 (SE 0.0118) [0.085; SE 0.0116] for hearing status and 0.039 (SE 0.0083) [0.039; SE 0.0083] for head patch.

Estimated genetic correlations among all traits were sizable and significantly different from 0 (*P* < .001). Between hearing status and number of blue eyes, the genetic correlation estimated using the observed (liability) scale was +0.566 (SE 0.0875) [+0.629; SE 0.0836]. Between hearing status and presence of head patch, the genetic correlation estimated using the observed (liability) scale was −0.865 (SE 0.0663) [−0.863; SE 0.0686]. Estimates of correlation of litter effects were smaller in magnitude, but similar in direction: +0.360 (SE 0.1135) and +0.330 (SE 0.1085) between hearing status and number of blue eyes on the observed and liability scales, respectively, and −0.296 (SE 0.1125) and −0.326 (SE 0.1113) between hearing status and head patch on the observed and liability scale, respectively.

### 3.3 Assessment of phenotypic and genetic trends in CSD

**FIGURE 1:**
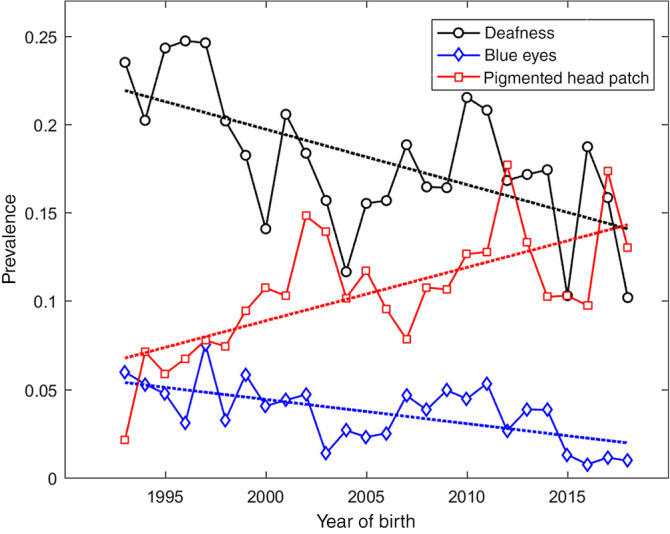
Prevalence of total congenital sensorineural deafness (CSD; uni‐ and bilateral) (“deafness,” black, circles), presence of blue eyes (blue, diamonds), and presence of a pigmented head patch (red, squares) by year of birth. Predicted values from regression (best fit line) are shown as dotted lines

The number of puppies with unilateral and bilateral CSD per year of birth, and the prevalence of unilateral, bilateral, and total CSD, per year from 1993 to 2018 are shown in Table 5. Regression of prevalence of total CSD (uni‐ and bilateral CSD) on year of birth indicated a significant downward trend (−0.0031 per year, *P* < .01, 95% confidence interval [CI] −0.0049 to −0.0014). This is equivalent to a decrease in prevalence of −0.0815 (or 8.15%; CI −12.75% to −0.04%) over the entire 26‐year period for which yearly data were complete (1993‐2018). For uni‐ and bilateral deafness individually, the regression of prevalence on year of birth also identified significant downward trends (−0.0018, *P* < .01 and −0.0014, *P* < .01), equivalent to decreases in prevalence of 4.6% and 3.6% over the 26‐year period, respectively. Similar analyses of changes in prevalence of the traits determined as genetically related to CSD, presence of blue eyes and presence of patch, yielded statistically significant trends in the direction indicated by the genetic correlations: −0.0014 per year (*P* < .001), a decrease of −3.6% over the 26‐year period for the presence of blue eyes, and 0.0030 (*P* < .001) and increase of 7.8% over the 26‐year period in the presence of patch. The prevalence of total CSD (uni‐ and bilateral), presence of blue eyes, and presence of a patch by year of birth are depicted in Figure 1.

**TABLE 5:** Prevalence of deafness over year of birth

Year of birth	Numbers of dogs				Prevalence		
	Bilateral hearing	Unilateral CSD	Bilateral CSD	Total BAER tested	Unilateral CSD	Bilateral CSD	Total (uni‐ + bilateral CSD)
1993	179	35	20	234	14.96%	8.55%	23.50%
1994	213	36	18	267	13.48%	6.74%	20.22%
1995	193	46	16	255	18.04%	6.27%	24.31%
1996	268	63	25	356	17.70%	7.02%	24.72%
1997	291	63	32	386	16.32%	8.29%	24.61%
1998	344	67	20	431	15.55%	4.64%	20.19%
1999	354	61	18	433	14.09%	4.16%	18.24%
2000	360	52	7	419	12.41%	1.67%	14.08%
2001	309	61	19	389	15.68%	4.88%	20.57%
2002	329	57	17	403	14.14%	4.22%	18.36%
2003	430	63	17	510	12.35%	3.33%	15.69%
2004	364	41	7	412	9.95%	1.70%	11.65%
2005	332	48	13	393	12.21%	3.31%	15.52%
2006	371	53	16	440	12.05%	3.64%	15.68%
2007	280	48	17	345	13.91%	4.93%	18.84%
2008	264	39	13	316	12.34%	4.11%	16.46%
2009	219	33	10	262	12.60%	3.82%	16.41%
2010	248	46	22	316	14.56%	6.96%	21.52%
2011	297	54	24	375	14.40%	6.40%	20.80%
2012	188	26	12	226	11.50%	5.31%	16.81%
2013	280	42	16	338	12.43%	4.73%	17.16%
2014	218	36	10	264	13.64%	3.79%	17.42%
2015	209	17	7	233	7.30%	3.00%	10.30%
2016	217	39	11	267	14.61%	4.12%	18.73%
2017	223	36	6	265	13.58%	2.26%	15.85%
2018	282	30	2	314	9.55%	0.64%	10.19%

*Notes:* By year of birth the number of puppies tested for brainstem auditory evoked response (BAER) in this data set, the number and prevalence of unilateral and bilateral congenital sensorineural deafness (CSD), and the total CSD prevalence. The total prevalence was used as the dependent *y*‐variable in the linear regression on year of birth to determine the phenotypic trend.

The regression of mean EBV (from univariate analysis of hearing status on the observed scale) on year of birth (1993‐2018) gives a coefficient similar in magnitude and also significantly different from 0; −0.0036 (*P* < .001; CI, −0.0042 to −0.0030), suggesting that genetic change is the driving force behind the decrease in the prevalence of CSD. This figure can be used as the observed response (*R*) to presumed selection. Rearranging the equation *R* = *i h* ^2^ *σ*_P_/*L* (where *L* is the approximate generation interval of 4 years) gives an estimate of *i* (the mean deviation of individuals with phenotypic values exceeding the truncation point^15^) as −0.091. The approximate selection intensity from the response observed is equivalent to excluding 4% to 5% with the highest genetic liability of all animals from breeding. Scaling up the regression coefficient gives an estimated genetic change over the 26‐year period (1993‐2018) of −0.0930, which is 32.5% of the additive genetic variance, and concordant with the difference between mean EBV in 1993 and 2018 born animals (−0.0935).

## 4 DISCUSSION

Our study includes data on the largest number of Dalmatian puppies undergoing BAER as screening for CSD reported to date, and suggests that CSD is a moderately heritable condition in the UK KC‐registered Dalmatian population. Furthermore, we found an improving trend in both phenotypes, by decreasing the prevalence of CSD, and genetic liability, using EBVs for CSD, over the last 20 to 30 years. The magnitude of the genetic improvement observed suggests that it is the principal driver of the decrease in phenotypes, which is not surprising given the previous lack of identified environmental influences that potentially could be managed to lower individual risk. This genetic improvement is most likely because of the incorporation of BAER screening results into breeding decisions, with the response equivalent to a selection intensity of avoiding the 4% to 5% of animals with the highest genetic risk across the whole population.

The overall prevalence of CSD in our study was 17.8% (13.4%, unilateral; 4.4%, bilateral), which is only slightly lower than the prevalence (18.4%; 13.1%, unilateral; 5.3%, bilateral) reported by the only other study on Dalmatians conducted in the UK, published in 1997.^4^ However, the period over which the data analyzed previously^4^ were gathered was the 4 years (1992‐1995) immediately after ‐initiation of the BAER testing scheme in 1992. It therefore provides a useful baseline against which to compare any response to selection that has occurred since the scheme's launch. The dogs included in the previous study^4^ were tested at 3 different institutions, 1 of which is the institution of the current study, and it is possible that some of the breeders involved in the current study also contributed data to the previous study.^4^ In our study, the data comprises BAER test results from 1992 to 2019 (although trends were determined over “complete” years, 1993‐2018), a much longer period than, but partly incorporating, that of the previous study.^4^ The prevalence of CSD over the most recent 4 full years included in our study (2015‐2018) was 13.72% (11.31%, unilateral; 2.41%, bilateral), which compared to the baseline of the previous study shows some notable improvement (ie, more than halving of the prevalence of bilateral deafness and a 25% decrease in overall prevalence), which is consistent with the improving phenotypic trend reported.

The heritability estimate of CSD in our study was moderate, at approximately 0.3 (across various models). This is large enough to elicit a response because of selection on phenotypes, as was observed and reported. The derived approximate selection intensity of 4% to 5% is consistent with excluding all bilaterally deaf dogs from breeding (assuming the prevalence in this data reflects that of the wider population). However, this derived selection intensity is equivalent to excluding 4% to 5% of all potential breeding dogs (ie, across the entire population), but it is unlikely that all selection decisions were influenced by BAER test results, as not all litters underwent or undergo BAER screening. To the best of authors’ knowledge, the Animla Health Trust (AHT) is the largest BAER testing center in the UK, and has been over the period these data encapsulate. The total number tested in this data set per year of birth (Table 5) as a proportion of total number registered by the KC increased from 10% in 1993 to 30% in 2018 (a significantly increasing trend of 0.7% per year, *P* < .001). If, as we expect, this trend is indicative of a general increase in participation in BAER testing, then it implies that any selection based on BAER testing will have been restricted to a minority of the breeding candidates in the overall population. This implies that the selection intensity applied by breeders actively participating in BAER testing exceeds that derived here (ie, that those participating in screening are using the results to make breeding decisions).

However, because the heritability is much <1, it means that selection will not be particularly accurate, because ranking according to genetic risk is not the same as ranking according to phenotype. This is because in a trait with only 3 ordinal phenotypic categories (bilaterally hearing, unilaterally deaf and bilaterally deaf), but with a presumed underlying continuous, normally distributed genetic risk, there will be considerable genetic variation in risk among dogs that have the same categorical phenotype (eg, bilaterally hearing). Even if all puppies were BAER tested and breeders then excluded all CSD dogs from breeding, doing so only would exert a selection intensity of <20% and still include many phenotypically unaffected but higher genetic risk animals. The routine provision of EBVs for CSD would have 2 benefits: (a) a continuous, quantitative metric of risk enabling discrimination between bilaterally hearing dogs with “high” and “low” genetic risk and (b) universality—they are available for all animals in the pedigree, even if they do not have a phenotype. Not only are EBVs a more accurate indicator of genetic risk, but because they are continuous (rather than categorical), they can facilitate the application of a higher selection intensity. However, there are administrative and logistic obstacles to the routine calculation of EBVs (eg, having to link unregistered dogs into KC pedigree).

The heritability estimates of CSD given here are consistent with those reported for Dalmatians and other breeds both in the UK and in other countries, although the range is large: 0.27 (Germany^7^), 0.57 (Switzerland^6^), 0.32 (USA^5^), and 0.75 (USA^9^) in Dalmatians, and in other breeds 0.31 in Jack Russell Terriers (USA^18^), 0.15 in English Bull Terriers (UK^13^), and 0.36 in Border Collies (UK^12^). However, estimates of heritability are population‐specific and thus may differ across breeds and countries because of either variations in the magnitude of additive genetic variance or non‐additive genetic variance (including environmental risk factors), which are both constituents of phenotypic variance.

In agreement with previous investigations,^5^, ^7^ our study identified a strong genetic association in the Dalmatian between CSD and blue irises, and a negative association between CSD and the presence of a pigmented head patch. The magnitude, as with heritability, will be population‐specific, depending on the additive genetic variance of each trait (and the covariance between them). Here, correlations of CSD on the observed scale were +0.57 with number of blue eyes, and −0.86 with presence of a head patch, consistent with those calculated in other studies of Dalmatians (+0.53 with blue eyes and −0.36 with presence of patches^7^; −0.53 with presence of a patch^5^) and other breeds (+0.58 with blue eyes in Border Collies^12^; −0.54 with head patch in English Bull Terriers^13^). Thus, it appears that there is a general association between CSD and blue eyes, and CSD and pigmentation phenotypes (absence of patches) in the Dalmatian and other dog breeds, which also has been reported in several other species, including cats,^11^, ^19^ horses,^11^ and possibly even in humans (eg, Waardenburg syndrome^20^). Although the details of the precise genetic causes and mechanism of the relationship between deafness and (absence of) pigmentation remain unknown, what can be determined from these genetic correlations is that selection for a decrease in the prevalence of deafness will elicit a correlated response of a decrease in the prevalence of blue eyes, and an increase in prevalence of head patches, as was observed in our study. However, the small estimates of heritability of head patches determined here (0.08) mean that the predicted correlated response (calculated according to equations previously outlined^15^) would be very small, at <4% increase in head patches when breeding only from the 50% of animals with the lowest genetic risk of CSD. This should not be at odds with any other selection objectives, and have no effect on success in the show ring in the UK because the KC breed standards explicitly state “some patching on ears or head not to be penalized” (https://www.thekennelclub.org.uk/services/public/breed/standard.aspx?id=4087). However, this is not the case in many other countries, including the United States. The change in prevalence of pigmented head patch observed here was larger than a correlated response would predict. This may be a consequence of some breeders selecting for (or easing selection against) pigmented patches on the understanding that it is genetically related to CSD, or it may simply be a result of random sampling of the non‐additive genetic factors affecting pigmented patches, which given the low heritability, will have greater proportional influence.

In conclusion, our study has provided further evidence that CSD as determined by BAER is moderately heritable (as has been previously reported on many occasions and in many breeds). However, our study also demonstrates, for the first time, improvement in terms of both a significantly decreasing trend in the prevalence of CSD over year of birth and a comparative decrease in the most recent estimate of the prevalence of CSD from baseline levels determined in UK KC‐registered Dalmatians born between 1992 and 1995. A corresponding decrease (improvement) in average genetic risk (EBVs) per year of birth indicates that this finding is most likely a consequence of selection against CSD. Genetic correlations between CSD and blue eyes, and CSD and pigmented head patches, imply that a correlated response in these traits would occur as a result of selection for a decrease in CSD prevalence, but that, in the case of pigmented head patches, this decrease would be very small. The regular calculation and public provision of EBVs for CSD from BAER testing would enable improvement in selection by (a) increasing the accuracy of selection (as compared to selection on phenotypes), and (b) by universal provision of a continuous, quantitative metric (rather than a categorical phenotype).

## CONFLICT OF INTEREST DECLARATION

T. L. is a full‐time employee of the Kennel Club. The other authors have no conflict of interest.

## OFF‐LABEL ANTIMICROBIAL DECLARATION

Authors declare no off‐label use of antimicrobials.

## INSTITUTIONAL ANIMAL CARE AND USE COMMITTEE (IACUC) OR OTHER APPROVAL DECLARATION

The study has been approved by the institution's Ethics committee.

## HUMAN ETHICS APPROVAL DECLARATION

Authors declare human ethics approval was not needed for this study.
